# Coronavirus disease 2019 (COVID-19) can predispose young to Intracerebral hemorrhage: a retrospective observational study

**DOI:** 10.1186/s12883-021-02109-8

**Published:** 2021-02-19

**Authors:** Michael T. Lawton, Ehsan Alimohammadi, Seyed Reza Bagheri, Arash Bostani, Siavash Vaziri, Ali Karbasforoushan, Kossar Mozaffari, Mehran Bahrami Bukani, Alireza Abdi

**Affiliations:** 1 Department of Neurological Surgery, Barrow Neurological Institute, St. Joseph’s Hospital and Medical Center, Phoenix, AZ USA; 2 Department of neurosurgery, Kermanshah University of Medical Sciences, Kermanshah, Iran; 3 Department of neurology, Kermanshah University of Medical Sciences, Kermanshah, Iran; 4 Infectious Disease Research Center, Kermanshah University of Medical Sciences, Kermanshah, Iran; 5 Department of anesthesiology, Kermanshah University of Medical Sciences, Kermanshah, Iran; 6 Clinical Research Development Center, Imam Reza hospital Kermanshah University of Medical Sciences, Imam Reza hospital, Kermanshah, Iran; 7 Nursing and midwifery school, Kermanshah University of Medical Sciences, Kermanshah, Iran

**Keywords:** Coronavirus disease 2019, Central nervous system, Intracerebral hemorrhage, chronic arterial hypertension, hematoma.

## Abstract

**Background:**

The respiratory system involvement is the most common presentation of Coronavirus disease 2019 (COVID-19). However, other organs including the central nervous system (CNS) could be affected by the virus. Strokes, seizures, change in mental status, and encephalitis have been reported as the neurological manifestation of the disease. We hypothesized that COVID-19 could predispose younger patients to spontaneous intracerebral hemorrhage (ICH). The present study aimed to investigate whether COVID-19 has any relationship with the occurrence of spontaneous ICH in young or not.

**Methods:**

We retrospectively evaluated all the patients with spontaneous ICH who were referred to our center between 20 Feb and 1 Sep 2020. The demographic, clinical, radiological, and laboratory test data were evaluated. Patients were divided into two groups. The COVID-19 positive patients and COVID-19 negative ones. All the variables including age, sex, history of hypertension, diabetes mellitus, smoking, Glasgow Coma Scale (GCS), hematoma volume and location, the presence of intraventricular hemorrhage and hydrocephalus on admission, the length of hospital stay, the lab test results and the clinical outcome at last visit or discharge as Glasgow Outcome Scale (GOS) were compared between the two groups.

**Results:**

There were 22 COVID-19 positive patients (20.8%) and 84 COVID-19 negative ones (79.2%). The mean age of the patients in the case group (54.27 ± 4.67) was significantly lower than that in the control group (69.88 ± 4.47) (*p* < 0.05). Meanwhile, our results showed a significant difference between the two groups based on the presence of chronic arterial hypertension (p < 0.05). There were no significant differences between the two groups based on gender, diabetes mellitus, smoking, Glasgow Coma Scale (GCS), hematoma volume, need for surgery, the presence of intraventricular hemorrhage and hydrocephalus on admission, White Blood Cell (WBC) count, platelet count, Prothrombin Time (PT), and Partial Thromboplastin Time (PTT) (*p* > 0.05).

**Conclusions:**

Our results show that COVID positive patients with ICH are younger and with less predisposing factors than COVID negative subjects with ICH.

## Background

Coronavirus disease 2019 (COVID-19) caused by the severe acute respiratory syndrome coronavirus 2 (SARS-CoV-2) that reported in December 2019 in Wuhan City, Hubei Providence, China for the first time [1].

The World Health Organization (WHO) declared COVID-19 as a global pandemic on March 11, 2020.

The typical manifestation of COVID-19 is respiratory system involvement. It can present as cough, shortness of breath, fever, chills, muscle pain, sore throat, fatigue, malaise, etc. [2]. However, the COVID-19 can affect the other organs of the body such as the central nervous system (CNS) [2, 3]. It has been reported that the disease could be associated with neurological presentations including seizures, change in mental status, and encephalitis [2]. Strokes have been reported as one of the neurological manifestations of the disease [4].

Stroke is the third reason for death in advanced countries. Intracerebral hemorrhage (ICH) constitutes about 10 to 15% of all strokes [5].

Spontaneous intracerebral hemorrhage is defined as bleeding within the brain parenchyma in the absence of underlying causative entities such as trauma, tumors, and vascular malformations [6]. Chronic arterial hypertension is the most common cause of spontaneous intracerebral hemorrhage as a result ICH occurs more in the elderly population [7].

Large-vessel stroke has been reported as a presenting feature of COVID-19 in the young [8]. We hypothesized that COVID-19 infection could be accompanied by spontaneous intracerebral hemorrhage in young in the absence of the predisposing chronic arterial hypertension. As a result, in this study, we aimed to investigate whether COVID-19 has any relationship with the occurrence of spontaneous ICH in young or not.

## Material and methods

This study evaluated all patients with spontaneous intracerebral hematoma (ICH) who admitted to Imam Reza Hospital, Kermanshah, Iran between 20 Feb and 1 Sep 2020. The present study was approved by the Scientific Research Board of the Kermanshah University of Medical Sciences.

Patients with a secondary intracerebral hemorrhage (ICH due to trauma, tumor, and vascular lesions) were not included. Meanwhile, those with a history of anticoagulant medications were excluded. All patients were new emergency department admissions. A brain computed tomography (CT) scan was performed on admission. A complete neurological examination was performed at the emergency department and twice daily after admission. The demographic, clinical, radiological, and laboratory test data were collected. A second brain CT scan was performed for patients with neurological deteriorations. The location of hematoma and hematoma volume was determined according to the initial brain CT scan of all patients. We calculated the hematoma volume according to the ellipsoid formula (4/3 π a × b × c), where a, b, and c represents the respective radii in 3-dimensional neuroimaging [9]. The location of hematoma was divided into four categories including lobar, deep, cerebellar, and brain stem. We divided patients into two groups. The COVID-19 positive group and the COVID-19 negative one. A SARS-CoV-2 infection has confirmed by nucleic acid-based polymerase chain reaction (PCR) and/or a positive chest high-resolution CT (HRCT) examination within 48 h after admission. We compared all the variables including age, sex, history of hypertension, diabetes mellitus, smoking, Glasgow Coma Scale (GCS), hematoma volume and location, the presence of intraventricular hemorrhage and hydrocephalus on admission, the length of hospital stay, the lab test results and clinical outcome at last visit or discharge as Glasgow Outcome Scale (GOS) between the two groups.

### Statistical analysis

The data analysis was performed using the SPSS 21 software (SPSS Inc. Chicago, Illinois).

Data are presented as mean ± standard deviation. The independent t-test, the Chi-square test, and the *Fisher’s exact test* were used to compare different variables between the two groups. *P* values < 0.05 were considered as the significant level.

## Results

There were 22 COVID − 19 positive patients (20.8%) and 84 COVID-19 negative ones (79.2%). (Table 1). Of a total of 22 COVID − 19 positive patients, 14 cases (63.63%) were asymptomatic at admission. Tables 1 and 2 show the descriptive statistics of the study.

**Table 1:** Frequency and frequency percent of the variables

Variable		Frequency	Frequency Percent
COVID-19	Positive	22	20.8
	Negative	84	79.2
Gender	Male	58	54.7
	Female	48	45.3
Hypertension	Yes	60	56.6
	No	46	43.4
Diabetes	Yes	30	28.3
	No	76	71.7
Smoking	Yes	26	24.5
	No	80	75.5
Hematoma Location	Lobar	36	33.9
	Deep	54	51
	Cerebellum	10	9.4
	Brain Stem	6	5.7
Focality of hematoma	Unifocal	91	85.84
	Multifocal	15	14.15
GOS	Death	22	20.8
	Vegetative State	10	9.4
	Sever Disability	22	20.8
	Moderate Disability	34	32.1
	Good Recovery	18	17.0
Need For Surgery	Yes	30	28.3
	No	76	71.7
Intera-Ventricular Hemorrhage	Yes	22	20.8
	NO	84	79.2
Hydrocephalus	Yes	14	13.2
	No	92	86.8

**Table 2:** Mean and standard deviation of quantitative variables

variable	Mean (SD)
Age (Year)	66. 64 (7.79)
GCS	7.84 (1.86)
Hospital stay (day)	16.43 (6.86)
Hematoma volume (CC)	17.33 (8.72)
WBC count	9360 (5404)
Platelet count	226,011 (98308)
Prothrombin Time	14.80 (1.22)
Partial Thromboplastin Time	32.03 (3.08)

**Fig. 1:**
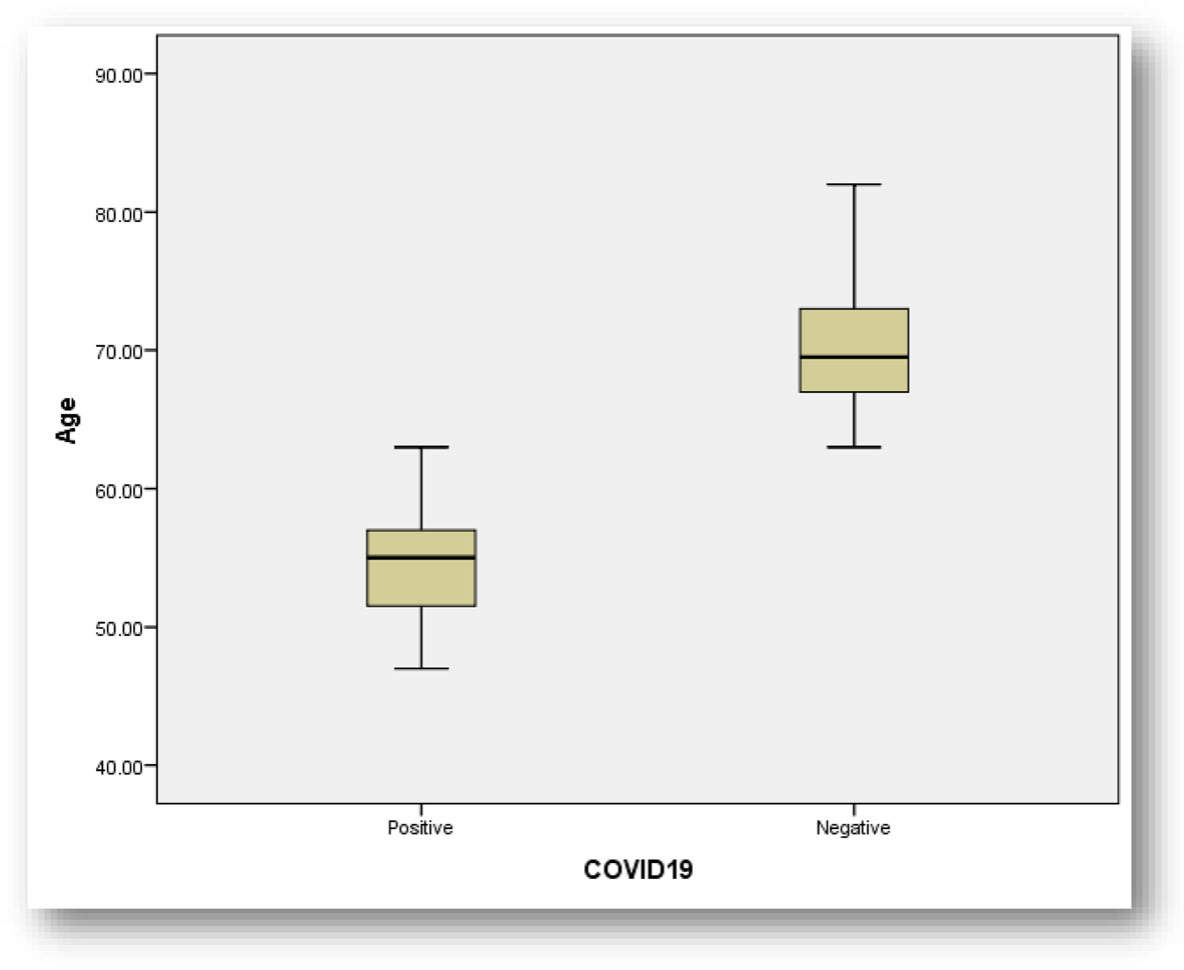
Comparison between the COVID-19 positive group and COVID-19 negative one based on the age

The mean age of the patients in the case group (54.27 ± 4.67) was significantly lower than that in the control group (69.88 ± 4.47) (*p* < 0.05) (Table 4) (Fig. 1).

Moreover, there was a significant difference between the two groups based on the presence of chronic arterial hypertension(*p* < 0.05) (Table 3).

**Table 3:** Comparing two groups (COVID positive and negative) in term of qualitative variables

Variable			COVID Positive (%)	COVID Negative (%)	Statistical test
Gender		Male	14 (24.1)	44 (75.9)	*P* = 0.502
		Female	8 (16.7)	40 (83.3)	
Hypertension			4 (6.7)	56 (93.3)	*P* = 0.006*
Diabetes			4 (13.3)	26 (86.7)	*P* = 0.482
Smoking			4 (15.4)	22 (84.6)	*P* = 0.711
Hematoma Location		Lobar	10 (27.7)	26 (72.3)	*P* = 0.866
		Deep	10 (18.51)	44 (81.48)	
		Cerebellum	4 (40.0)	6 (60.0)	
		Brain Stem	0 (00.0)	6 (100.0)	
Focality of hematoma		Unifocal	13 (14.2)	78 (85.8)	*P* = 0.07
		Multifocal	9 (60.0)	6 (40.0)	
GOS	Unfavorable outcome	Death	10 (45.5)	12 (54.5)	*P* = 0.03*
		Vegetative State	2 (20)	8 (80)	
		Sever Disability	4 (18.2)	18 (81.8)	
	Favorable outcome	Moderate Disability	4 (11.8)	30 (88.2)	
		Good Recovery	2 (11.1)	16 (88.9)	
Need for Surgery			8 (26.7)	22 (73.3)	*P* = 0.708
Intera-Ventricular Hemorrhage			8 (36.4)	14 (63.6)	*P* = 0.210
Hydrocephalus			4 (28.6)	10 (71.4)	*P* = 0.625

(*) means *p* < 0.05

Our results showed no significant differences between the two groups in term of gender, diabetes mellitus, smoking, Glasgow Coma Scale (GCS), hematoma volume, need for surgery, the presence of intraventricular hemorrhage and hydrocephalus on admission, WBC count, platelet count, PT, and PTT (*p* > 0.05) (Tables 3, 4).

**Table 4:** Relationship between COVID-19 status and quantitative variables

variable	Positive Mean (SD)	Negative Mean (SD)	Statistical test
Age (Year)	54.27 (4.67)	69.88 (4.47)	*P* < 0.001*
GCS	8.36 (1.43)	7.71 (1.95)	*P* = 0.308
Hospital stay (day)	22.01 (5.89)	14.97 (6.38)	*P* = 0.002*
Hematoma volume (CC)	19.81 (10.40)	16.69 (8.25)	*P* = 0.295
WBC	8590 (2343)	95.61 (59.58)	*P* = 0.601
Platelet count	261,363 (88962)	216,752 (99519)	*P* = 0.183
PT	14.48 (1.41)	14.88 (1.16)	*P* = 0.331
PTT	31.45 (3.20)	32.19 (3.07)	*P* = 0.487

(*) means *p* < 0.05

The mean length of hospital stay was significantly higher for the cases in the case group (22.01 ± 5.89) in comparison with that in the control group (14.97 ± 6.38) (*p* < 0.05) (Table 4).

The COVID-19 positive group had a worse outcome than the COVID-19 negative ones based on GOS (Table 3).

## Discussion

Our results showed that COVID-19 can lead to ICH in young in the absence of predisposing factors including chronic arterial hypertension.

Chronic arterial hypertension is the main underlying cause of spontaneous intracerebral hemorrhage [6]. Long-lasting uncontrolled arterial hypertension leads to the damage of blood vessels with a diameter of 50 to 200 μm through muscular wall hypertrophy, endothelial lining damage, and lipohyalinosis [10, 11]. These damages lead to the weakening of the blood vessels wall that presents as Charcot-Bouchard aneurysms [11]. The rupture of these truly arteriolar dissections is the leading cause of the majority cases of hypertensive intracerebral hemorrhage [10, 11].

There are some hypotheses that may justify the occurrence of intracerebral hemorrhage in patients with COVID-19 in the absence of chronic arterial hypertension.

The COVID-related coagulopathy presenting as dysfunctional homeostasis and cytokine storm mediated endothelitis and vasculitis of the CNS have been suggested as the possible mechanisms of ICH in these patients [12]. SARS-CoV-2 could trigger CNS vasculitis, possibly through an inflammatory response mediated by the cytokine storm [13, 14].

Meanwhile, COVID-19-associated intracerebral hemorrhage has been described to also exhibit attributes of demyelination [15] Moreover, underlying endothelial reactivity, as well as endothelial and neuropil degeneration has been reported in COVID positive patients with ICH [16].

Some studies have suggested viral infections like influenza as a stroke trigger [17, 18]. Influenza could aggravate the pathophysiology of stroke [19]. The cytokine storm induced by viral infections including influenza A and COVID-19 could be considered as an explanation for the occurrence of strokes in the affected patients [19, 20]. It has been shown that these viral infections could lead to systemic inflammation with a high level of IL-6, IL-1β, and TNFα [19, 20]. As shown in the previous studies, this systemic inflammation can lead to the breakdown of collagen and alteration in the permeability of the blood-brain barrier (BBB) [19]. Moreover, it has been reported that Influenza A virus infection could lead to elevation of matrix metalloproteinase-9 (MMP-9) and as a result a collagen breakdown in the basal membrane of the arterial walls. Interestingly, such mechanisms have been suggested for the instability of the wall of the intracranial aneurysms [21].

Muhammad et al. in a mouse model study demonstrated the influenza virus infection could lead to the release of inflammatory cytokines such as RANTES [19]. This circulatory cytokine could induce a higher expression of macrophage inflammatory protein-2 (MIP-2) in the BBB cells. MIP-2 recruits inflammatory cells such as neutrophil to the ischemic brain parenchyma. Neutrophils could mediate the ischemic brain damage through the releasing of MMP-9 which degrades the extracellular matrix of the BBB. Moreover, high plasma levels of MMP-9 which have been seen in influenza virus-infected mice could be considered as the trigger of the ICH [19].

Another explanation that may justify the neurological manifestation of the disease is a direct invasion of the virus in the brain. It has been shown that the brain tissue had been edematous and hyperemic during the autopsy of patients with COVID-19 [4].

The virus, similar to other respiratory viruses such as severe acute respiratory syndrome coronavirus (SARS-CoV) and Middle East respiratory syndrome coronavirus (MERS-CoV), could enter the central nervous system through the hematogenous or retrograde neuronal route [4].

Moreover, the severe acute respiratory syndrome coronavirus nucleic acid has been detected in the cerebrospinal fluid of some affected patients as well as it has been found in brain tissue of affected patients on autopsy [4, 22, 23].

Our results showed that the mean age of COVID-19 positive patients (54.27 ± 4.67) was significantly lower than the mean age of the control group (69.88 ± 4.47). Although, some studies reported that the neurological complications of COVID-19 infection including encephalitis, olfactory, and gustatory disturbances are occurred more in elderly patients [24].

Li et al. in a retrospective study of data from the COVID-19 outbreak in Wuhan, China, reported.

an incidence of about 5% for stroke among hospitalized patients with Covid-19. The youngest subject in their study was 55 years old [24]. However, Oxley et al. reported five.

COVID-19 positive patients younger than 50 years of age who presented with a large-vessel stroke [8].

### Limitations

The present study has several limitations. This study was a retrospective analysis of a single-center experience and all data were extracted from the electronic medical records of all patients. The small sample size was another important limitation of this work that could cause biases in clinical observation and could limit the generalizability of our findings. Furthermore, we assessed the clinical outcome only at discharge or last visit. Moreover, in order to effectively establish a causal effect of COVID-19 towards intracerebral hemorrhage, large population studies that evaluate the risk of intracerebral hemorrhage comparatively in cohorts of COVID-19 and non-COVID-19 patients are necessary. In such studies, the use of age as a co-founding factor may reliably elucidate a potential triggering effect of COVID-19 towards intracerebral hemorrhage in the young. So, some large, multicenter prospective trials would be needed for a better evaluation of the possible predisposing role of COVID-19 for ICH.

## Conclusions

Our results show that COVID positive patients with ICH are younger and with less predisposing factors than COVID negative subjects with ICH.

## Data Availability

The datasets generated and/or analysed during the current study are not publicly available due them containing information that could compromise research participant privacy/consent but are available from the corresponding author on reasonable request. All data are available from the corresponding author upon reasonable request. The patient’s data included in this manuscript has not been previously reported.

## Abbreviations

COVID-19: Coronavirus disease 2019
CNS: Central nervous system
ICH: Intracerebral hemorrhage
GCS: Glasgow Coma Scale
GOS: Glasgow Outcome Scale
WBC: White Blood Cell
PT: Prothrombin Time
PTT: Partial Thromboplastin Time
PCR: Polymerase chain reaction
HRCT: High-resolution computed tomography
BBB: Blood-brain barrier
MMP-9: Matrix metalloproteinase-9
MIP-2: Macrophage inflammatory protein-2
SARS-CoV: Severe acute respiratory syndrome coronavirus
MERS-CoV: Middle East respiratory syndrome coronavirus
